# Renal arterial pseudoaneurysm after robotic-assisted partial nephrectomy: a single-center analysis

**DOI:** 10.1007/s00345-025-05851-7

**Published:** 2025-09-02

**Authors:** Yu-Pin Huang, Hsiao-Jen Chung, I-Shen Huang, Tzu-Ping Lin, Shing-Hwa Lu, Eric Y. H. Huang

**Affiliations:** 1 Department of Urology, Fu Jen Catholic University Hospital, Fu Jen Catholic University, New Taipei City, Taiwan; 2 Department of Urology, Taipei Veterans General Hospital, No. 201, Section 2, Shipai Road, Taipei, 11217 Taiwan; 3 Department of Urology, College of Medicine and Shu-Tien Urological Research Center, National Yang Ming Chiao Tung University, Taipei, Taiwan

**Keywords:** Renal tumor, Robotic-assisted partial nephrectomy, Renal artery pseudoaneurysm, Radius exophytic/Endophytic nearness anterior/Posterior location nephrometry score, Preoperative aspects and dimensions used for an anatomical score

## Abstract

**Purpose:**

To determine the incidence of renal artery pseudoaneurysm (RAP) after robotic-assisted partial nephrectomy (RaPN), identify predictive factors, and evaluate endovascular management.

**Methods:**

The institutional RaPN database was retrospectively reviewed, and data from December 2009 to June 2021 were used. Computed tomography angiography was used to diagnose RAP. Patients who underwent embolization for RAP after RaPN were compared with those without RAP or with RAP managed conservatively. Data on patient demographics, tumor characteristics, and operative outcomes were evaluated, and the predictive factors for RAP after RaPN were determined.

**Results:**

Of the 544 patients who underwent RaPN, 14 developed RAP after surgery, of which 12 underwent embolization. Most patients experienced gross hematuria and were diagnosed using computed tomography angiography. No patient exhibited recurrent RAP during follow-up. The following was found for patients who underwent embolization for RAP: a higher proportion of men (91.7% vs. 59.4%, *p* = 0.024), higher RENAL nephrometry scores (median: 9.0 vs. 8.0, *p* = 0.02), longer operative times (mean: 349.6 vs. 283.7 min, *p* = 0.046), and longer postoperative hospital stays (median: 6.0 vs. 5.0 days, *p* = 0.031). The N score in the RENAL nephrometry score was significantly higher in the embolization group (*p* = 0.031) than in the nonembolization group. Univariate analysis revealed that RENAL nephrometry scores and total operative time were significant predictors of RAP occurrence.

**Conclusion:**

The occurrence of RAP was associated with higher RENAL nephrometry scores and longer operative times.

## Introduction

Renal cell carcinoma is a common urological malignancy worldwide. Epidemiological data from the United States have revealed an increasing incidence of renal cell carcinoma over recent decades [1]. With the increased application of imaging modalities, the occurrence of incidentally detected renal cell carcinoma has increased [2]. For T1a/T1b renal masses, partial nephrectomy (PN) is the standard treatment [3, 4]. Although PN is technically more challenging than radical nephrectomy, its oncologic outcomes are similar, and it preserves renal function better than radical nephrectomy [5, 6]. Advances in minimally invasive techniques and robotic surgical platforms, such as the da Vinci robotic system, provide a three-dimensional field and enable delicate wrist actions and a greater degree of flexibility. Therefore, robotic-assisted PNs (RaPNs) are widely performed. RaPNs decrease blood loss and the duration of hospital stays compared with open PN [7]. Nevertheless, a serious life-threatening complication after PN is renal artery pseudoaneurysm (RAP), which, however, has a low incidence [8, 9]. RAP usually occurs in the early postoperative period and typically presents with gross hematuria, flank pain, and anemia [9]. Emergent transarterial embolization is an effective treatment for hemodynamically unstable RAP patients [10, 11].

The anatomy of renal masses and complexity of PN can be quantified using two scoring systems: (1) the RENAL nephrometry score, which evaluates tumor radius (R), exophytic/endophytic properties (E), nearness to the collecting system or sinus (N), anterior/posterior location (A), and location relative to polar lines (L) and (2) the preoperative aspects and dimensions used for an anatomical (PADUA) score, which reflects anatomical complexity [12, 13]. These tools are used to assess the complexity of planned surgeries for renal tumors and guide surgeons’ management decisions. The risk factors for RAP occurrence have been studied; however, no consensus has been reached on whether the level of renal tumor complexity affects RAP occurrence [8, 14–16]. Furthermore, not all patients with RAP after PN require interventions, and some patients can be treated conservatively [17]. This study aimed to identify predictive factors specifically associated with RAP cases requiring embolization to improve the understanding and management of clinically significant postoperative pseudoaneurysms after RaPN.

## Patients and methods

### Patients

A prospectively maintained database was retrospectively reviewed to identify patients who underwent RaPN for renal tumors between December 2009 and June 2021. This observational study was approved by the Institutional Review Board (TPEVGH-IRB 2023-02-001BC) and conducted per the Declaration of Helsinki and the ICH-GCP guidelines. The requirement for informed consent was waived by the IRB because of the retrospective nature of the study and the anonymization of patient data.

### Surgical procedures

RaPN was performed using a transperitoneal or retroperitoneal robotic-assisted technique, with vascular control achieved per surgeon’s preference. Tumor excision was consistently performed with a margin of normal renal parenchyma to ensure oncologic safety, and pure enucleation was not routinely performed. Renorrhaphy was performed after tumor excision in a stepwise manner. Transected vessels and collecting system defects at the resection site base were closed using running 3–0 V-Loc sutures. Next, vascular clamps were released to restore blood flow and ensure adequate hemostasis. Hemostatic agents, such as Floseal, were applied over the defect, and a surgical bolster was placed. Finally, the edges of the renal parenchyma were approximated using 1−0 Polysorb sutures (Covidien), an absorbable synthetic copolymer made of glycolide and lactide (Lactomer 9−1).

### RAP diagnosis and management

RAP was diagnosed via computed tomography (CT) angiography for patients with clinical signs of RAP, such as hematuria, flank pain, and anemia, after RaPN or during the follow-up protocol. Decisions regarding whether patients underwent embolization for RAP were based on the patients’ clinical conditions. Transarterial angioembolization was performed according to the interventional radiology protocol.

Patients were routinely followed up in the outpatient clinic 10–14 days after discharge to review pathology and assess postoperative recovery. Follow-up imaging was typically recommended for asymptomatic patients every 3 months during the 1st postoperative year, with ultrasound and CT alternated based on physician preference and clinical context. CT renal angiography was not routinely performed at the 1- or 3-month follow-up unless prompted by symptoms suggesting RAP, such as gross hematuria, flank pain, and unexplained anemia. Considering this was a retrospective study, the actual follow-up intervals varied between patients. Follow-up duration was defined as the time between surgery and the last available outpatient visit or imaging at our institution.

### Data collection

Patients were categorized into two groups for evaluating the factors associated with clinically significant RAP requiring intervention: those who underwent transarterial embolization for RAP (the embolization group) and those who did not, including patients without RAP and those managed conservatively (the nonembolization group). Patient demographics and baseline characteristics included the following: gender, body mass index, American Society of Anesthesiologists physical status classification, use of antiplatelet or anticoagulant, tumor size, tumor RENAL nephrometry score [12], tumor PADUA classification [13], operative time, estimated blood loss, ischemia time, and intraoperative complications (according to Clavien–Dindo classification). The tumor size was defined as the maximal diameter (in mm) determined by the preoperative CT scan. The pathological diagnosis was made according to the 2004 World Health Organization classification.

### Statistical analysis

Descriptive statistics of the categorical variables are reported as means and standard deviations or medians and interquartile ranges. Continuous variables were compared using the Mann–Whitney U test, and categorical variables were compared using the χ^2^ test. Univariate logistic regression models were used to determine the variables that independently correlated with RAP occurrence. Statistical analyses were performed using SPSS for Windows version 22 (IBM, Armonk, NY, USA). A two-sided *p* value of < 0.05 was considered statistically significant.

## Results

A total of 544 patients who underwent RaPN for renal tumors were included in this study. Of these, 14 (2.6%) developed RAP, 12 (2.2%) underwent embolization, and 2 were managed conservatively. Of 12 patients who underwent embolization, 10 and 2 received transperitoneal RaPN and retroperitoneal RaPN, respectively. The median time to RAP presentation was 9.5 days. Two patients had a history of antiplatelet or anticoagulant use; both had these medications withheld before surgery.

Of two patients managed conservatively, one had a 6-mm RAP detected during hospitalization for flank pain. Follow-up CT at 6 months showed resolution of the lesion, with no recurrence observed on annual imaging for up to 4 years. The other patient had a 9-mm RAP with perirenal hematoma detected during hospitalization. CT at 6 months showed no residual RAP, with no recurrence observed on follow-up imaging over 2 years.

The embolization group had a significantly higher proportion of male patients (91.7% vs. 59.4%, *p* = 0.024) and RENAL nephrometry scores (median 9.0 vs. 8.0, *p* = 0.02) than the nonembolization group, with only the N component being significantly higher in the embolization group. No significant differences in PADUA scores were observed between the groups (Table 1).

**Table 1:** Patient and tumor characteristics

Variables	Embolization (*n* = 12)	No embolization (*n* = 532)	*p* value
Age (years), mean ± SD	58.9 ± 9.0	57.1 ± 13.2	0.654
Male (%)	11 (91.7%)	316 (59.4%)	0.024
BMI, median (IQR)	26.4 (25.1–28.3)	25.2 (22.7–27.8)	0.154
ASA, median (IQR)	2.0 (2.0–2.0)	2.0 (2.0–2.0)	0.770
Right side (%)	8 (66.7%)	286 (53.8%)	0.375
Tumor size on CT (mm), median (IQR)	39.0 (33.5–43.0)	36.0 (27.0–50.0)	0.739
RENAL nephrometry score, median (IQR)	9.0 (8.0–9.0)	8.0 (7.0–9.0)	0.02
Radius	1.5 (1.0–2.0)	1.0 (1.0–2.0)	0.606
Exophytic/endophytic	2.0 (1.0–2.0)	2.0 (1.0–2.0)	0.2
Nearness to collecting system or sinus	3.0 (3.0–3.0)	3.0 (2.0–3.0)	0.031
Anterior (%)	7 (58.3%)	286(53.9%)	0.446
Location relative to polar lines	2.5 (2.0–3.0)	2.0 (1.0–3.0)	0.227
PADUA score, median (IQR)	10.0 (9.0–11.0)	9.0 (8.0–10.0)	0.073
Polar location	2.0 (1.0–2.0)	2.0 (1.0–2.0)	0.355
Exophytic	2.0 (1.0–2.0)	2.0 (1.0–2.0)	0.192
Renal rim	1.0 (1.0–2.0)	1.0 (1.0–2.0)	0.989
Renal sinus invasion	2.0 (2.0–2.0)	1.0 (1.0–2.0)	0.075
Collecting system invasion	2.0 (1.0–2.0)	1.0 (1.0–2.0)	0.168
Tumor size	1.5 (1.0–2.0)	1.0 (1.0–2.0)	0.771

*ASA* American Society of Anesthesiologists physical status classification, *BMI* body mass index, *CT* computed tomography, *IQR* interquartile range, *PADUA* preoperative aspects and dimensions used for an anatomical, *RENAL* radius exophytic/endophytic nearness anterior/posterior location

The operative time and postoperative hospital stay were significantly longer in the embolization group than in the nonembolization group (349.6 vs. 283.7 min, *p* = 0.046 and 6.0 vs. 5.0 days, *p* = 0.031, respectively). No significant differences in other surgical parameters, including warm ischemia time, estimated blood loss, and intraoperative blood transfusion rates, were observed between the two groups (Table 2).

**Table 2:** Surgical outcomes and functional follow-up data

Variables	Embolization (*n* = 12)	No embolization (*n* = 532)	*p* value
Operative time (min), mean ± SD	349.6 ± 113.1	283.7 ± 91.4	0.046
Warm ischemia time (min), median (IQR)	29.5 (22.2–34.3)	23.0 (16.0–32.0)	0.109
EBL (ml), median (IQR)	287.5 (200.0–462.5)	180 (100.0–350.0)	0.077
Intraoperative transfusion (%)	2 (13.3%)	46 (8.6%)	0.679
Postoperative stays (days), median (IQR)	6.0(5.0–7.0)	5.0 (5.0–6.0)	0.031
Time to RAP (days), median (IQR)	9.5 (5.0–13.5)		
Follow-up period (months), median (IQR)	23.0 (20.5–52.5)	26.0 (9.0–58.0)	0.565

*EBL* estimated blood loss, *IQR* interquartile range, *RAP* renal artery pseudoaneurysm

**Table 3:** Patient, tumor, and surgery characteristics and clinical presentations in individual patients diagnosed with renal artery pseudoaneurysm (RAP)

Patient no.	Treatment		Gender	Age	Tumor size (cm)	Warm ischemia time (min)	Tumor pathology	Clinical presentation	Postoperative days to symptoms	Operation	RENAL nephrometry score	PADUA score	Antiplatelet or anticoagulant use
1	Embolization		M	48	36	22.3	AML	Flank pain	10	Transperitoneal	113a1	111,111	No
2	Embolization		M	63	37	17	Clear cell	Flank pain, gross hematuria	9	Transperitoneal	123a3	322,221	Yes
3	Embolization		M	61	34	23	Chromophobe	Gross hematuria	10	Transperitoneal	123p3	222,221	No
4	Embolization		M	65	43	53	Clear cell	Gross hematuria	31	Transperitoneal	223a1	121,212	No
5	Embolization		M	55	47	31	Clear cell	Gross hematuria, urinary retention	41	Transperitoneal	223p3	221,222	No
6	Embolization		M	52	41	30	Chromophobe	Gross hematuria, urinary retention	15	Transperitoneal	223p2	121,212	Yes
7	Embolization		M	56	32	25	Clear cell	Flank pain, gross hematuria	5	Transperitoneal	123a3	221,221	No
8	Embolization		M	71	42	28.9	Oncocytoma	Gross hematuria	13	Transperitoneal	223a3	222,222	No
9	Embolization		M	58	17	22	Clear cell	Flank pain, gross hematuria	9	Retroperitoneal	133p2	231,211	No
10	Embolization		F	73	64	43	AML	Flank pain	5	Transperitoneal	313p2	212,112	No
11	Embolization		M	42	43	41	Clear cell	Flank pain	5	Transperitoneal	223a2	221,222	No
12	Embolization		M	63	32	17	Clear cell	Gross hematuria	5	Retroperitoneal	113a3	211,121	No
13	Conservative treatment		M	40	25	14	Clear cell	Flank pain	4	Transperitoneal	133a3	231,111	No
14	Conservative treatment		M	47	46	17	Leiomyoma	Flank pain	5	Retroperitoneal	213p1	112,112	No

Treatment refers to the clinical management approach for RAP. “Embolization” indicates patients who underwent angiographic embolization. “Conservative treatment” indicates patients managed without intervention

*AML* angiomyolipoma, *PADUA* preoperative aspects and dimensions used for an anatomical, *RENAL* radius exophytic/endophytic nearness anterior/posterior location

Univariate analysis revealed that higher RENAL nephrometry scores and longer total operative time significantly predicted RAP occurrence and the need for embolization.

Table 3 shows the demographic and tumor characteristics and clinical presentations of the 14 patients diagnosed with RAP (12 who underwent embolization and 2 who were managed conservatively). Most patients presented with gross hematuria and were diagnosed using contrast-enhanced CT. All embolization procedures successfully controlled bleeding, and none of the patients required nephrectomy or experienced rebleeding during follow-up. The median follow-up duration was 23 months, and no patients were lost to follow-up.

## Discussion

PN is beneficial for pT1a and even pT1b lesions owing to oncological control and preservation of renal parenchyma [5, 6]. Minimally invasive technology is advancing quickly, and RaPN is a favorable treatment option for nephron-sparing surgery. Nevertheless, RaPN is challenging and may lead to complications, including RAP. The incidence of RAP after minimally invasive RaPN is higher than that after the open approach to PN [7, 11, 18, 19]. Risk factors for RAP were identified in several studies [8, 16, 17, 20, 21]. However, few studies have focused on pseudoaneurysm in patients undergoing RaPN [17]. The present study demonstrated that the occurrence of RAP after RaPN is low (2.6%) and that higher RENAL nephrometry scores and longer total operative times are predictors for RAP occurrence.

Tumor complexity may influence RaPN outcomes and RAP occurrence; however, the results of previous studies are controversial. In a study by Omae et al. [16], CT angiography was performed 3–4 days after minimally invasive PN in 101 patients, and univariate analysis revealed that RAP was associated with large tumor size, a higher N component score, renal sinus exposure, and opening of the collecting system. Our study showed that the RENAL nephrometry score N component was higher in the embolization group than in the nonembolization group; however, univariate analysis revealed that only higher RENAL nephrometry scores were associated with RAP occurrence. This discrepancy may be due to the unbalanced patient numbers in the two groups. Conversely, the renal sinus invasion component of the PADUA score did not differ between the two groups, which is not in agreement with the findings of Omae et al. Excision and suturing of the renal parenchyma and sinus may lacerate the arterioles, resulting in RAP; however, this was not directly reflected in the occurrence of RAP.

Herein, the incidence of RAP after RaPN was 2.6%. A systematic review by Jain et al. [9] reported an incidence of 1.00% for open PN and 1.96% for minimally invasive approaches, including laparoscopic and robotic PN, indicating a significant difference between the two techniques (*p* < 0.001). In Chavali et al.’s [8] study, the incidence rate of RAP was 1.4% in 1,417 patients who underwent PN, and univariate analysis revealed that longer operative time and cold ischemia time were significant risk factors for RAP. In our study, high RENAL nephrometry scores and long total operative times were associated with RAP occurrence. The differences between these two studies is potentially because of different study populations: Chavali et al. analyzed patients who underwent minimally invasive or open PNs, whereas our study only included patients who underwent RaPN. Furthermore, RaPN in patients with higher RENAL nephrometry scores is more challenging. Besides, RENAL nephrometry scores were higher in the embolization group in our study than those in Chavali et al.’s (9 vs. 8) study. This may explain why high RENAL nephrometry scores were a risk factor only in our study.

Tumor location may influence the occurrence of complications after PN. Nadu et al. [20] defined central tumors as enhancing lesions coming into contact with or invading the collecting system or renal sinus. In their study of 212 patients who underwent laparoscopic PN, including 53 patients with central tumors, Nadu et al. found that RAP was significantly more common in patients with central tumors [20]. Additionally, the proximity of the tumor to the collecting system was associated with overall surgical complication rates in a study by Sempels et al. [22], which is consistent with our study.

Our results demonstrated that higher RENAL nephrometry scores, but not PADUA scores, were associated with RAP occurrence. This difference may be due to the differently classified tumor complexities of the groups. For the embolization group, RENAL nephrometry scores indicated intermediate complexity, whereas PADUA scores indicated high complexity. Furthermore, the N component differs between the RENAL nephrometry and PADUA score systems. The N component includes the collecting system and renal sinus involvement in the RENAL nephrometry scoring system, whereas the collecting system and renal sinus involvement are considered separately in the PADUA scoring system. Overall, nephrometry scores are useful because they provide surgeons with preoperative information. However, the occurrence of complications depends not only on nephrometry scores but also on the surgeon’s experience, surgical plan, and patient comorbidities.

The present study has several strengths. All patients analyzed in this study underwent RaPN. Thus, the occurrence of RAP on the robot platform was revealed. Our study also analyzed RENAL nephrometry scores, PADUA scores, and their nephrometry components simultaneously, providing a more comprehensive analysis of RAP risk factors. Nevertheless, there are several limitations to the present study. First, this was a retrospective study, which may have been influenced by confounders. Second, all patients were analyzed from a single institute, which may not reflect interinstitute and general population differences. Third, not all operations were performed by a single surgeon, and different surgeons may have different experience levels and techniques. Additionally, the long study period may have introduced variability in surgical techniques and perioperative care owing to refinements and improvements over time. Finally, the low occurrence of RAP and the unbalanced distribution between groups, combined with the retrospective nature of the study and the absence of an a priori sample size calculation, may have limited the statistical power of certain comparisons.

In conclusion, the incidence of RAP after RaPN was 2.6% at our institute. The occurrence of RAP was associated with higher RENAL nephrometry scores and longer total operative times. Hence, for patients with higher RENAL nephrometry scores and longer operative times, postoperative vigilance for RAP occurrence is required.

## Data Availability

No datasets were generated or analysed during the current study.
